# RCA 1-binding glycans as a marker of *Batrachochytrium salamandrivorans* infection intensity at early stages of pathogenesis

**DOI:** 10.1038/s41598-025-21554-w

**Published:** 2025-10-28

**Authors:** So Jeong Yoon, Elin Verbrugghe, Eduardo Fernández Meléndez, Sofie De Bruyckere, Ellen Blomme, Diederik Strubbe, Léa Fieschi-Méric, Frank Pasmans, An Martel

**Affiliations:** 1 Wildlife Health Ghent, Department of Pathology, Pharmacology and Zoological Medicine, Ghent University, 9820 Merelbeke, Belgium; 2 Research Institute for Nature and Forest (INBO), Havenlaan 88 bus 73, 1000 Brussels, Belgium; 3 Terrestrial Ecology Unit, Ghent University, KL Ledeganckstraat 35, 9000 Ghent, Belgium

**Keywords:** Chytridiomycosis, *Batrachochytrium salamandrivorans*, RCA 1, Galactose, Early stage infection buildup, Lectin histochemistry, Biochemistry, Microbiology

## Abstract

The fungal skin disease chytridiomycosis threatens global amphibian biodiversity. Among the two causative pathogens, *Batrachochytrium dendrobatidis *and *B. salamandrivorans*, the latter has decimated several European salamander populations. However, responses to exposure vary notably across species and individuals, suggesting that understanding the mechanisms behind these variations could enable successful mitigation in surviving populations. A recent study reported that the concentration of epidermal galactose predicts *B. salamandrivorans* susceptibility at the species level. Here, we used comparative lectin histochemistry with nine lectins to observe lectin staining brightness in the skin and examine its association with the susceptibility of four urodelan species with differential responses to *B. salamandrivorans*. Subsequently, we exposed *Pleurodeles waltl *individuals to *B*. *salamandrivorans* to assess whether this correlation extends to the individual level. In our study, the degree of labelling with the lectin, Ricinus communis agglutinin I (RCA 1), emerged as the distinctive marker of susceptibility in that resistant species showed minimal RCA 1 binding, while susceptible species exhibited higher levels. Results of the infection trial further demonstrated that an increase in staining brightness correlates with steeper infection slopes at initial stages of infection. This demonstrates RCA 1-binding glycans as a biomarker for individual susceptibility and presents the basis for marker-assisted selection in *P. waltl*.

**Supplementary Information:**

The online version contains supplementary material available at 10.1038/s41598-025-21554-w.

## Introduction

The decline of global amphibian populations is driven by disease and human-induced threats such as climate change and habitat loss^1−3^. Specifically, locations where species are transported beyond their native ranges due to human activities have exacerbated these challenges by the introduction of novel pathogens into naive populations. Long-term coevolution can lead to coexistence between hosts and pathogens, where species become resistant or tolerant to infection^4^. However, various factors like the aforementioned threats can disrupt this balance resulting in deadly outcomes^5^. Emerging infectious diseases refers to such diseases appearing in new geographic areas or populations, and to existent infections rapidly surging in incidence or expanding their geographic range^6^. In particular, the devastating impact of the global amphibian panzootic caused by chytridiomycosis exemplifies how emergent pathogens can lead to an irreversible collapse in biodiversity.

The causative agents of the chytridiomycosis skin disease are two chytrid fungi, *Batrachochytrium dendrobatidis *(*Bd*) and *B*.* salamandrivorans *(*Bsal*), which invade the skin and impair vital physiological functions, leading to severe consequences in susceptible species^7−9^. The more recently described, *Bsal*, was discovered a decade ago in a population of fire salamanders (*Salamandra salamandra*) in the Netherlands^7^ and has since been detected in neighbouring countries, spanning from Belgium and Germany to as far as Spain^10^. The origin of the pathogen can be traced back to Asia^8,11^ with the spread to Europe suggested to have resulted from the poorly regulated amphibian trade^8,12^.

Although strategic preventative measures such as trade restrictions, pre-import screening tests, and biosecurity protocols for fieldwork^13^ may be effective^14^, it is critical to develop solutions that target the pathogen when prevention fails, as well as long-term management plans and conservation protocols that facilitate the recovery of depleted populations. Clinical treatments have been developed for *Bsal*^15^ but given the pathogen’s ability to persist in situ despite changes in the environment and decreasing host numbers^16^, post-treatment reintroduction is not an efficient option.

An extensive review of published literature and infection records from field surveys and captive settings by Monzon et al. (2023) revealed that 67 species from 10 different amphibian families tested positive for *Bsal *across 69 countries. Notably, *Bsal*-related deaths were reported in 29 species exclusively from the Salamandridae and Plethodontidae families^17^. Although *Bsal *has not been recorded outside Asia and Europe thus far, North American species are potentially highly susceptible hosts of the disease^8,18,19^. Furthermore, anurans may not be mere carriers of the pathogen^20^ since they can also develop clinical signs of chytrid after experimental exposure^19^.

Given the urgency in containing the spread, it is critical to deepen our understanding of its fundamental biology and epidemiology. As part of the prioritisation strategy^14,21^, research has focused on identifying host susceptibility^8,18,19,22,23^ and regional vulnerability^24−27^, to predict the risk and impact of *Bsal* incursion into naive populations. However, to support the recovery of already affected communities, it is crucial to characterise the mechanisms by which certain hosts exhibit resistance to *Bsal*. Elucidating the factors underlying variation in response to exposure requires a thorough understanding of the complex interplay between the pathogen and its host during pathogenesis. One significant knowledge gap concerning chytrids in general is the characterisation of the physical host-parasite interface and the molecules involved in adhesion^28^. Pathogen adherence to host epithelial surfaces marks the beginning of the colonisation process, and can also trigger the expression of virulence factors^29,30^. This adherence is mediated by the glycoconjugates present at the surface of host cells, formed by the covalent attachment of carbohydrates to other biomolecules such as proteins or lipids^31^. Surface adherence is primarily studied through glycan-binding proteins, which attach to the sugar residues of epithelial glycoconjugates. These are further classified into lectins and sulfated glycosaminoglycan-binding proteins^32^.

A recent study demonstrated the chemotaxis of *Bsal* zoospores towards several different saccharides. However, binding to specifically galactose in skin sloughs triggered a virulent response marked by the upregulation of proteolytic enzymes that degrade the skin and extracellular matrix^33^. This suggests that epidermal galactose facilitates the invasion process of these intracellular parasites. Using lectin histochemistry and peak infection loads as a proxy of susceptibility, the authors established a link between $\beta$-galactose-binding RCA 1 lectin signals and species vulnerability. More specifically, species resistant to *Bsal *showed little to no binding signals in the epidermis, while highly susceptible species exhibited intense staining. This represented a novel application of RCA 1 lectin histochemistry in identifying a marker for *Bsal *susceptibility across various species.

However, it is important to recognise that although lectins are often described as being specific to particular monosaccharides, they show higher preference for more complex sugar chains. Thus, their interactions with oligosaccharides that end in the same monosaccharide can vary across different lectins^34−37^. It is therefore essential to analyse the complete structure by utilising lectins with overlapping specificity for simpler sugars, but differing affinities for oligosaccharides. In this current study, we explored staining patterns of a selection of plant and recombinant prokaryotic $\alpha$- and $\beta$-galactoside-binding lectins and their suitability to be used as biomarkers for *Bsal *susceptibility at the species level.

In addition, a variation in glycosylation patterns within species has been reported^33^. This intraspecific variation in susceptibility is particularly important as it constitutes the foundation for the potential evolution of resistance and tolerance mechanisms^38,39^. If this epidermal glycosylation is a trait correlated with individual responses to *Bsal* exposure, it may be exploited to implement marker-assisted selection. Such an approach could contribute significantly to conservation efforts that aim to recover from the impact of *Bsal *by maintaining captive assurance populations, conferring an additional fitness advantage (i.e., higher resistance to the disease). Thus, to further examine whether the variation in the glycan content is correlated with *Bsal* susceptibility at the individual level, we conducted experimental exposures in *Pleurodeles* *waltl*, thereby assessing the trait’s potential for future breeding studies.

## Methods

### Animals

 Sampling and experimental exposures were conducted in accordance with European law and received approval from the ethical committee of the Faculty of Veterinary Medicine at Ghent University (EC2015/86; EC2023/59). All procedures adhered to relevant guidelines and regulations, including the ARRIVE (Animal Research: Reporting of In Vivo Experiments) guidelines, as outlined by NC3Rs (National Centre for the Replacement, Refinement and Reduction of Animals in Research, 215 Euston Road, London). Only captive-bred animals were utilised in this study.

### In vivo infection trial

 Captive-bred *P. waltl *(*n *= 59) were obtained from three private breeders (Table 1). Each individual was placed in plastic boxes containing moist paper towels and a plastic hide to ensure humidity and provide refuge, with the temperature maintained at 15 °C. To ensure full recovery before exposure, animals were abdominally implanted with a 9 mm passive integrated transponder tag (*Datamars, Spain*), weighed, measured snout-to-vent (SVL), and tail clipped 76 days before inoculation. Tail tissues were immediately processed and stained with RCA 1 following the method described below. Animals were exposed to *Bsal *for 24 hours with a 1 ml zoospore solution of 8.8 × 10^3^ spores/ml cultured from the *Bsal *type strain (AMFP 13/01)^7^. The progression of the disease was monitored through daily health checks and weekly skin swabs were taken to determine the *Bsal *load. Eight newts were kept uninfected as negative controls and underwent sham treatment. Swab samples were collected by stroking each specimen’s ventral side 10 times with a sterile rayon swab (*MWE, Corsham, UK)*. Pathogen load was then quantified from each swab using the real-time PCR assay described by Blooi et al. (2013). The experiment was concluded eight weeks post-exposure whereupon animals were weighed, measured, and underwent heat treatment for ten days at 25 °C to clear *Bsal *infection^40^.

**Table 1:** Infection Trial Animals.

Source	No. of individuals	Life stage	Mean weight (g) (*SD*)	Mean SVL (mm) (*SD*)
A	14	Adult	9.35 (±2.03)	57.24 (±4.92)
B	32	Adult	9.14 (±2.05)	58.15 (±4.32)
C	13	Adult	10.85 (±1.57)	61.58 (±4.26)

### Lectin screening

 Tissue samples from the dorsal tail of *Salamandra salamandra *(*n* = 6), *Pleurodeles waltl *(*n* = 6), *Ichthyosaura alpestris *(*n* = 6), and *Lissotriton helveticus *(*n* = 6) were used to screen lectin binding patterns. For each staining procedure, tissues from *Alytes obstetricans* and *S. salamandra *from a previous study^33^ were employed as negative and positive controls.

There are no anatomical topology differences in carbohydrate pattern between different body sites (i.e. ventral and dorsal skin, toe clips, and tail clips)^33^ but for standardisation and means of eliminating stressful anaesthesia procedures, 10 mm skin samples from the dorsal tail tip of each individual were collected using a sterile razor and fixed in 1 ml solutions of Bouin for 24 h. They were then paraffin-embedded and cut into 5 ${\mu }$m sections. Given the focus on keratinocyte-associated glycoproteins, rather than mucus preservation, tissues were processed using a standard paraffin-clearing protocol by deparaffinisation in xylene followed by hydration in a descending ethanol bath. After submersion in citrate buffer (10 mM citric acid, pH 6.0), samples were placed in a microwave for heat-induced antigen epitope retrieval. Following washing in phosphate-buffered saline (PBS, 0.01 M, pH 7.6), all sections were incubated for 15 min in 1.0% bovine serum albumin (BSA) within a wet box to block nonspecific binding and subsequently washed in PBS.

Based on the affinity of *Bsal* zoospores to terminal galactose^33,41^, five $\beta$-gal binding lectins were chosen and four $\alpha$-gal binding lectins were selected for comparison. The selection of fluorescein-conjugated lectins included, RCA 1 (*Ricinus communis *agglutinin I), ECL (*Erythrina cristagalli *lectin), GSL 1 (*Griffonia simplicifolia *lectin I), and PNA (Peanut (*Arachis hypogaea*) agglutinin) which were acquired from Vector Laboratories (*Burlingame, CA, US*).

Additionally, plant lectins can suffer from batch-to-batch variability during extraction and lower purity due to the presence of different isoforms^42^. To address these issues, we obtained lectins produced in bacterial systems through recombinant methods, which have also been reported to exhibit enhanced binding activity compared to their plant-derived counterparts. Five recombinant prokaryotic galactose-specific lectins were purchased from GlycoSeLect (*Dublin, IE*) and their secondary antibody, 6x-His Tag Monoclonal Antibody (HIS.H8), Alexa Fluor $^\textrm{TM}$ 488 from Thermo Fisher Scientific (*Waltham, MA, US*). A full list of all lectins used in the study is presented in Table 2.

For lectin histochemistry with plant lectins, prepped tissues were incubated in the dark within a wet box for 30 min with fluorescein-conjugated lectins diluted to 25 ${\mu }$ g/ml concentrations (exc. RCA 1= 15 ${\mu }$g/ml) with lectin binding buffer (10 mM HEPES, 0.15 M NaCl, PH 7.5) and later washed in PBS. For labelling using recombinant prokaryotic lectins (RPLs), the lectins were first diluted to a concentration of 20 ${\mu }$g/ml in Tris-buffered saline (TBS) supplemented with metal ions (0.05 M Tris, 0.15 M NaCl, pH 7.6, 1 mM CaCl2, 1 mM MnCl2, 1 mM MgCl2) and left at room temperature for 30 min for lectin-metal ion complex formation. Tissues were incubated in the dark within a wet box for 1 h with the diluted lectin solution and washed twice in PBS. The samples were subsequently incubated for 1 h with secondary antibody diluted to 10 ${\mu }$g/ml with TBS and washed twice in PBS.

**Table 2:** Plant & Recombinant Prokaryotic Lectins (RPLs).

Product No.	Origin	Lectin	Sugar specificity	Preferred structure	Plant Lectin equivalent	Inhibiting sugar
VEC.FL-1081	Plant	RCA 1	$\beta$-Gal, Lac^44^	Gal$\beta$1-4GlcNAc^45^	−	Gal / Lac
VEC.FL-1071-5	Plant	PNA	$\beta$-Gal^46^	Gal$\beta$1-3GalNAc^47^	−	Gal
VEC.FL-1141	Plant	ECL	$\beta$-Gal, GalNAc, Lac^48^	Gal$\beta$1-4GlcNAc^45,49^	−	Lac
VEC.FL-1101-2	Plant	GSL 1	$\alpha$-Gal^50^	$\alpha$1-3Gal^51^	−	Gal + GalNAc
L-001-2mg	Recombinant	RPL-$\alpha$Gal	$\alpha$-Gal & GalNAc	−	GSL-I	Gal + GalNAc
L-002-2mg	Recombinant	RPL-Gal1	$\beta$-Gal & LacNAc	−	ECL, RCA	Gal / Lac
L-003-2mg	Recombinant	RPL-Gal2	$\alpha$-Gal >GalNAc	−	GSL-I	Gal + GalNAc
L-004-2mg	Recombinant	RPL-Gal3	$\alpha$-Gal	−	GSL-IB4	Gal + GalNAc
L-005-2mg	Recombinant	RPL-Gal4	$\beta$-Gal & LacNAc & Lewis X	−	ECL, RCA	Gal / Lac

### Staining intensity scoring

 To limit the biases that skew post-analytical variables, the scoring system implemented was aligned with the key principles described by Gibson-Corley et al.^52^ allowing a more select conversion of the subjective perception of staining results into quantitative data. Lectin-stained tissues were scored blindly by four independent reviewers assigning individual brightness scores to each of the skin structures, following the methodology described by Wang et al.^33^, but applied per epidermal layer: (1) stratum corneum, (2) stratum spinosum, and (3) stratum *g*erminativum. By distinguishing the reactivity in each layer, a higher resolution of interaction patterns may be uncovered. A mean of the scores from each reviewer was taken as the final result. The staining intensities were classified as follows: negative = 0, weak = 1, strong = 2, and intense = 3. Tissues from the infection trial individuals subjected exclusively to RCA 1 staining, also underwent blind evaluation by four independent reviewers. Given that RCA 1 scores were rather consistent across the epidermal layers (Supplementary Table 3), the tissues were scored for the overall brightness of the epidermis and a mean value of these scores was taken as the final result. Staining intensities for RCA 1 were classified as follows: negative = 0, weak = 1, strong = 2, and intense = 3.

### Statistical analysis

 Statistical analyses were performed using R version 4.3.2^53^, with graphics generated using the ggplot2 package^54^. Kruskal-Wallis and post-hoc Dunn’s tests were used to compare RCA 1 scores of the present study to scores that Wang et al.^33^ attributed to the same species. The same tests were used to compare RCA 1 staining intensity scores across different skin layers.

To examine the relationship between glycan concentration and susceptibility, we used a metric that captures the rate of infection build-up in relation to the *Bsal* peak load within the first four weeks post-exposure. This metric describing “early stage infection buildup” corresponds to what previous studies have referred to as “infection intensity”^55,56^. The experimental animals used for the lectin screenings were not exposed to *Bsal*, so a direct statistical relationship could not be established. Instead, a descriptive analysis was performed to examine the species-level pattern between skin glycan concentration and susceptibility. Early stage infection buildup was estimated using *Bsal* load data from previous infection studies involving (*S. salamandra*,* I. alpestris*, and* L. helveticus*)^20^ and the infection trial of the present study (*P. waltl*). To test whether RCA 1 and other lectins scores are correlated, we built linear mixed models using the lme4 package^57^ with RCA 1 score as the response variable, with other lectin scores and skin type as fixed effects, and with species as a random factor, followed by correction for multiple hypothesis testing using the Bonferonni procedure. To determine whether epidermal glycosylation had an effect on early stage infection buildup in *P. waltl*, a linear mixed model was built with early stage infection buildup as the response variable, RCA 1 staining intensity score as the fixed effect, and source population as the random effect. Assumptions of homoscedasticity, linearity, and normality were verified by plotting models’ residuals against predicted values, and using a Shapiro-Wilk test with W $\ge$ 0.90, respectively.

### Sulfated glycotope detection

 Following the validation of RCA 1-glycans as a marker of species vulnerability, we employed three monoclonal antibodies (Table 3)—Anti-6-sulfo LacNAc Antibody, clone AG105 (A3251, *TCI Chemicals, Japan*), Anti-Keratan Sulfate Antibody, clone 5D4 (MABN248, *Merck Millipore*), and Anti-Glycosaminoglycan Antibody, clone PG4 (MABT819, *Merck Millipore*)—to detect the presence of specific sulfated glycan structures and their potential role in pathogen adhesion.

Of the three, the first detects a sulfated form of type-II LacNAc, the remaining two specifically target sulfated structures associated with glycosaminoglycans (GAGs). Cell surface proteoglycans, which possess GAG structures, frequently act as coreceptors for microbial pathogens, aiding in their interaction with secondary internalisation receptors. The fine structure of these GAGs is critical in the regulation of ligand-binding activities^58^.

Here we focused on GAGs, keratan and dermatan sulfate. Keratan sulfate chains contain a mixture of nonsulfated (3Gal$\beta$1-4GlcNAc$\beta$), monosulfated (3Gal$\beta$1-4GlcNAc6S$\beta$), and disulfated (3Gal6S$\beta$1-4GlcNAc6S$\beta$) disaccharide units^59^. These may occur in interstitial connective tissues but are also associated with various epithelial tissues including keratinocytes^60^. Dermatan sulfates are composed of repeating disaccharide units of either iduronic acid (IdoA) or glucuronic acid (GlcA) linked to *N-*acetyl galactosamine (GalNAc). These sugar residues can be esterified by sulfate groups at various positions along the disaccharide backbone^61^. In mammalian tissues, dermatan sulfates are the predominant glycan present in the skin and are also released at high concentrations during wound repair^62,63^.

The respective secondary antibodies of the above selection were as follows: Goat anti-Mouse IgM (Heavy chain) Cross-Adsorbed Secondary Antibody, Alexa Fluor$^\textrm{TM}$ 488 (*Thermo Fisher Scientific*), Goat anti-Mouse IgG1 Cross-Adsorbed Secondary Antibody, Alexa Fluor$^\textrm{TM}$ 568 (*Life Technologies*), and Goat anti-Mouse IgG, IgM (H+L) Secondary Antibody, Alexa Fluor$^\textrm{TM}$ 488 (*Thermo Fisher Scientific*).

**Table 3:** Monoclonal antibodies.

Generic name	Target antigen	Principle constituent disaccharide units
Anti-6-sulfo LacNAc Antibody (AG105)	6-sulfo LacNAc &	Gal($\beta$1,4)-GlcNAc(6S)($\beta$1,3)-^64^ &
	6-sulfo Lewis X	Gal($\beta$1,4)-[Fuc($\alpha$1,3)-GlcNAc(6S)($\beta$1,3)]-^64^
Anti-Keratan Sulfate Antibody (5D4)	Keratan Sulfate	[GlcNAc($\beta$1,4)-Gal($\beta$1,3)]*n*^65^
Anti-Glycosaminoglycan Antibody (PG4)	Dermatan Sulfate	[IdoA($\alpha$1,3)-GalNAc($\beta$1,4)]*n*^66^

For immunohistochemistry using the above antibodies, the tissues were processed according to the same methods as the lectin screening and then incubated overnight at room temperature with primary antibodies diluted 1:10 with PBS. Following a 20 min wash in PBS, samples were subjected to incubation with secondary antibodies, diluted 1:750 with TBS.

For all staining procedures, a negative control—treatment with inhibiting sugars and omission of the primary antibody before secondary antibody application—was included to demonstrate the specificity of the binding. Final tissues were mounted with ProLong Gold Antifade Mountant with DAPI (P36931, *Thermo Fisher Scientific*) and examined on the same day with an immunofluorescence microscope under 20× magnification, with the excitation maximum at 495 nm and the emission maximum at 515 nm. Images were taken with Leica Application Suite (*Leica Microsystem*) and further processed in ImageJ version 1.54^67^.

## Results

### Species-specific lectin binding patterns

#### $\beta$-**galactose binding lectins**

 The application of RCA 1 (Gal$\beta$1-4GlcNAc) resulted in positive staining of the keratinocyte surface of the stratum corneum, the cytoplasm of the cells in the stratum spinosum and stratum germinativum, and the underlying dermis in all species excluding *L. helveticus *and *A. obstetricans*. There was noticeable variation between the four species, with *S. salamandra *displaying the highest mean staining intensity score of 2.31 (± 0.38), followed by *I. alpestris *with 1.42 (± 0.92), *P. waltl *with 0.58 (± 0.52), and *L. helveticus *with 0.46 (± 0.37) in the stratum corneum. This trend was consistent across the stratum spinosum and stratum germinativum with only *L. helveticus *displaying scores below 1 in all three layers. Thus, only *Bsal*-susceptible and lethal species displayed consistent staining with RCA 1.

Both ECL (Gal$\beta$1-4GlcNAc) and PNA (Gal$\beta$1-3GalNAc) showed little to no reactivity in the epidermal layers across all species. For ECL-labelling, mean scores of the epidermis were below 0.50, similar to the negative control. PNA resulted in comparable stainings to ECL, although they were limited to the dermal glands with the exception of *A. obstetricans,* in which the stratum corneum was also stained.

The majority of individuals of all species displayed minimal concentration of RPL-Gal1 ($\beta$-gal and LacNAc) in the stratum corneum*,* with reactivity limited to the lower epidermal layers and dermis. In the stratum spinosum, mean scores for each species were (from smallest to largest): *L. helveticus,* 0.85 (± 0.26); *S. salamandra,* 0.89 (± 0.27); *I. alpestris,* 0.90 (± 0.41); and *P. waltl,* 1.44 (± 0.53). In the stratum germinativum, the scores were: *S. salamandra,* 0.91 (± 0.30); *L. helveticus,* 0.92 (*± *0.26); *I. alpestris,* 1.04 (± 0.48); and *P. waltl,* 1.63 (*± *0.50). Across all three strata, *P. waltl *consistently displayed the highest brightness intensity scores. Mean scores of RPL-Gal4 ($\beta$-gal and LacNAc, LeX) labelled stratum corneum were 1.60 (± 0.62) in *S. salamandra;* 1.48 (± 0.41) in *P. waltl;* 0.85 (± 0.67) in *I. alpestris;* and 0.56 (± 0.30) in *L. helveticus*. The stratum spinosum appeared non-reactive in a few individuals with mean scores below 1 in all species while the stratum germinativum exhibited an approximate score of 0.95 (± 0.20).

**Fig. 1:**
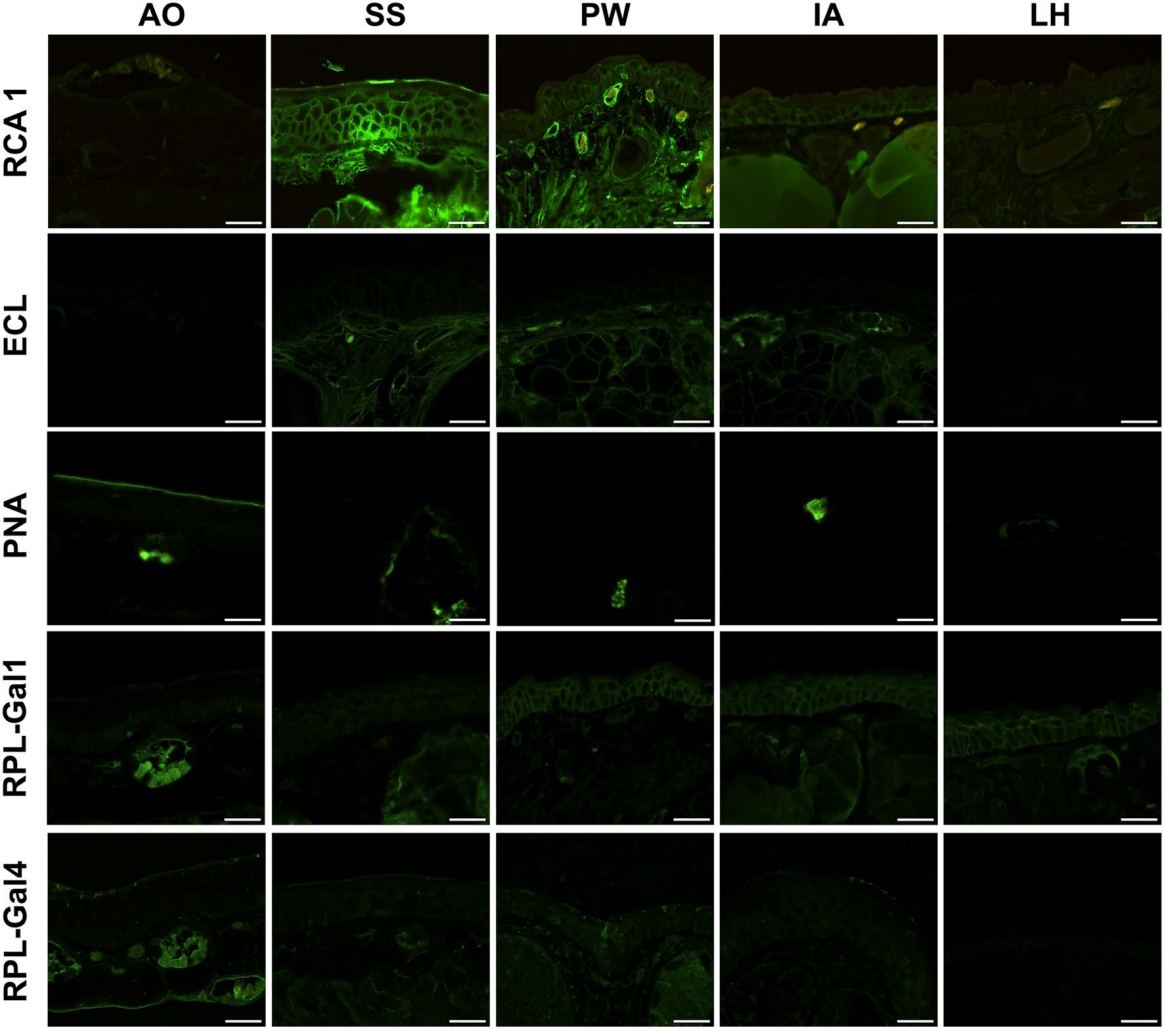
$\beta$-Gal lectins: RCA 1, ECL, PNA, RPL-Gal1, and RPL-Gal4 labelling results. Note the non-reactivity of the epidermis with PNA and ECL and faint signals of the recombinant prokaryotic lectins. AO, *A. obstetricans*; SS, *S. salamandra*; PW, *P. waltl*; LH, *L. helveticus*; IA, *I. alpestris*. Scale bar = 50 ${\mu }$m.

Figure 1 shows the binding patterns of each lectin by species and Supplementary Table 1 provides an overview of the mean staining intensity scores for $\beta$-binding lectins across each species which are visually presented in Fig. 3.

#### $\alpha$-**galactose binding lectins**

**Fig. 2:**
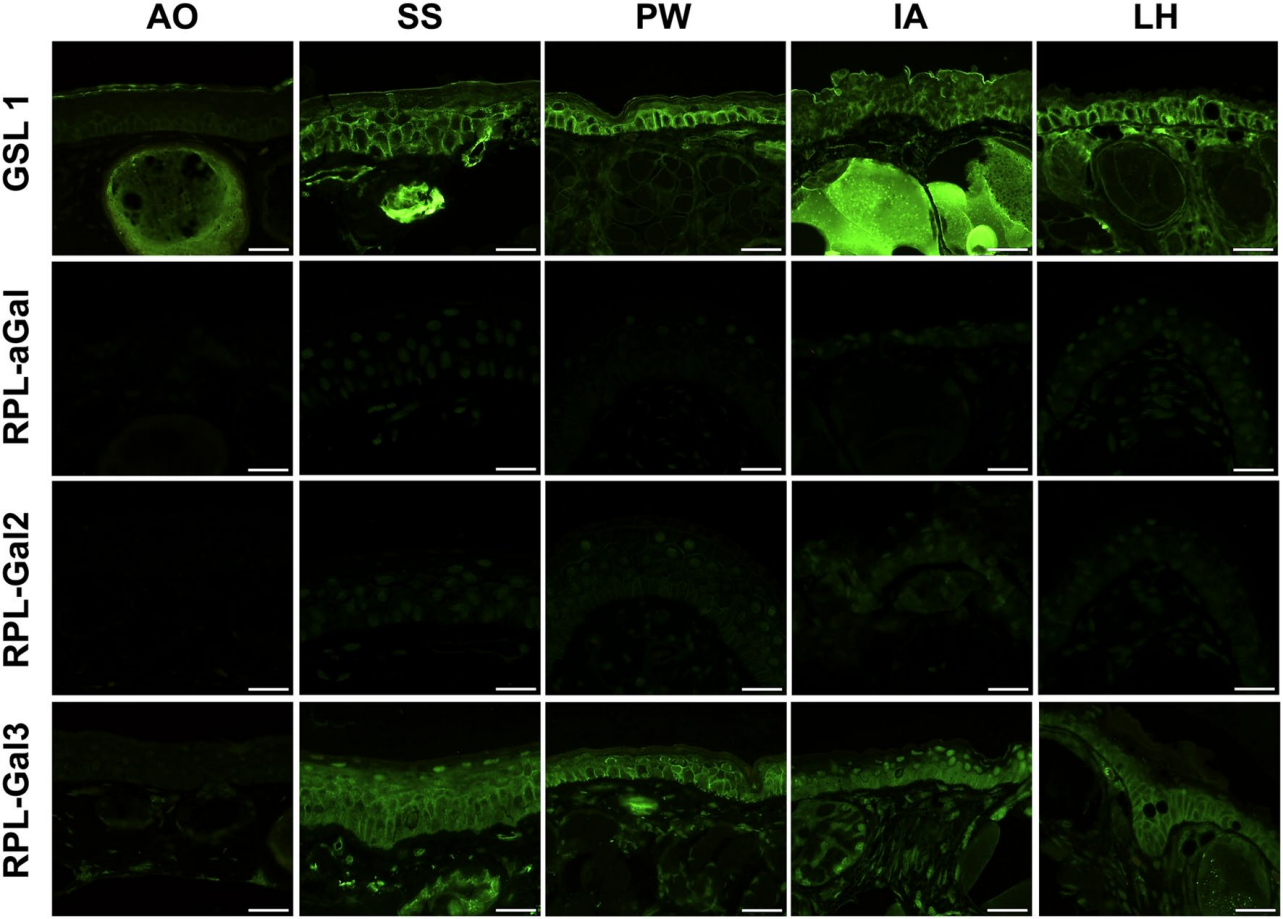
$\alpha$-Gal lectins: GSL 1, RPL-$\alpha$Gal, RPL-Gal2, and RPL-Gal3 labelling results. AO, *A. obstetricans*; SS, *S. salamandra*; PW, *P. waltl*; LH, *L. helveticus*; IA, *I. alpestris*. Scale bar = 50 ${\mu }$m.

**Fig. 3:**
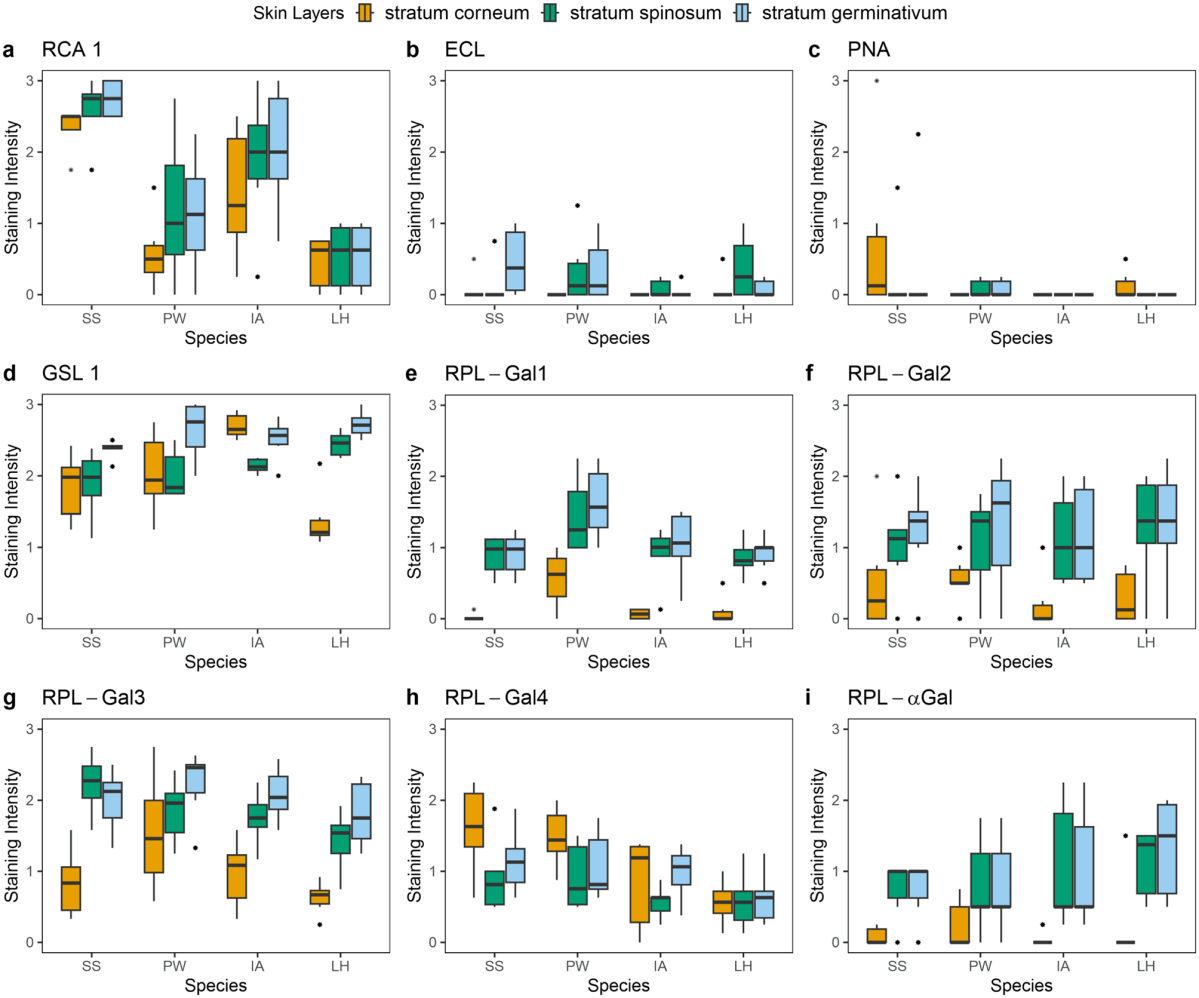
Boxplots depicting the distribution of staining intensity scores by species and skin layer of each lectin. Staining intensities were classified as follows: Negative = 0, weak = 1, strong = 2, and intense = 3. SS, *S. salamandra*; PW, *P. waltl*; LH, *L. helveticus*; IA, *I. alpestris*. Colours represent the different epidermal layers.

 GSL 1 presented a diffuse pattern in the epidermis across all species with the exception of *A. obstetricans *which exhibited reactivity in only the *s*tratum corneum and glands. Specifically in the stratum corneum, *L. helveticus *displayed the lowest mean staining score, 1.38 (*± *0.40); followed by *S. salamandra,* 1.85 (*± *0.46); *P. waltl,* 2.03 (*± *0.57); and *I. alpestris,* 2.70 (*± *0.17). The stratum spinosum and stratum germinativum yielded comparable scores across the four species, with average values of 2.12 (*± *0.21) and 2.56 (*± *0.13), respectively. RPL- $\alpha$ Gal ($\alpha$-gal, GalNAc) and RPL-Gal2 ($\alpha$-gal>GalNAc) presented near negative stainings. For RPL-Gal3 ($\alpha$-gal), cells of the epidermal layer and the dermal structures beneath exhibited clear staining in all species except the negative control, resembling the results of GSL 1. Mean intensity scores decreased among species as follows: *S. salamandra,* 2.23 (*± *0.41); *P. waltl,* 1.85 (*± *0.44); *I. alpestris,* 1.75 (*± *0.37); and *L. helveticus,* 1.43 (*± *0.41). The same order persisted in the underlying basal layer. However, like all previous $\alpha$-gal RPLs, the stratum corneum remained largely unstained across species with only *P. waltl *exhibiting a score exceeding 1. Figure 2 demonstrates the various binding patterns of each lectin by species. Supplementary Table 2 provides an overview of the mean staining intensity scores visually presented in Fig. 3.

### Predictors of species susceptibility

Susceptibility to *Bsal* is classified as (1) resistant, i.e., lack of infection and disease; (2) tolerant, i.e., infection but no disease; (3) susceptible, i.e., infection and disease; and (4) lethal, i.e., infection and disease leading to mortality^8^. Previous categorisation of the four species and additionally the negative control, a *Bsal*-resistant anuran, *A. obstetricans* are presented in Table 4, as well as mean RCA 1 scores^33^, average pathogen loads, and outcomes of infection.

**Table 4:** Species mean epidermal glycosylation and susceptibility to *Bsal *according to published literature.

	Mean Max logGE load (*SD*)	Mortality	Mean RCA 1 score (*SD*)^33^	Susceptibility	References
*Salamandra salamandra*	3.08 (±0.78)	100%	3.0 (±0.0)	Lethal	^8,10,20,33,68^
*Pleurodeles waltl*	3.05 (±0.69)	61.54%	2.1 (±0.4)	Susceptible	^8,10,33^
*Ichthyosaura alpestris*	2.90 (±1.02)	75%	2.7 (±0.3)	Susceptible	^8,20,33^
*Lissotriton helveticus*	0.32 (±0.78)	0%	0.6 (±0.3)	Resistant	^8,33^
*Alytes obstetricans*	0.44 (±0.68)	0%	0.4 (±0.5)	Resistant	^8,20,33^

#### Lectin histochemistry

**Fig. 4:**
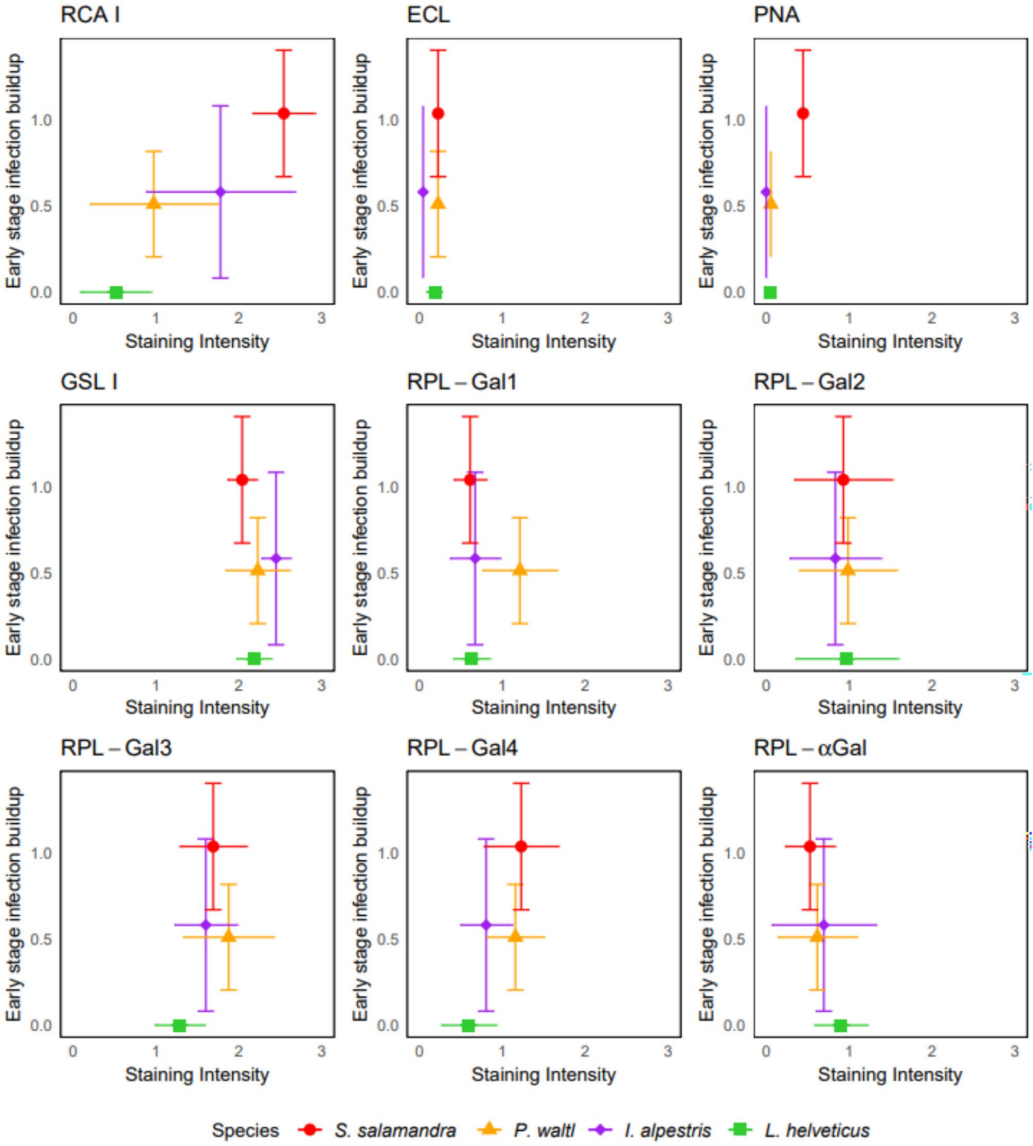
Relationship between mean staining intensity scores of the three epidermal strata and average early stage infection buildup of the different species. Early stage infection buildup was measured as the log10-transformed peak pathogen load divided by the latency (time in weeks) to reach the peak within the first four weeks following Bsal exposure. Colours and symbols represent the different species.

 Using the mean staining intensity scores across the three epidermal layers obtained above, our descriptive analysis revealed only RCA 1 distinctly differentiates species susceptibility as estimated through the early stage infection buildup index (Fig. 4). Thus, resistant species (low early stage infection buildup) display minimal staining whereas susceptible species (high early stage infection buildup) show higher levels of RCA 1-binding glycans. Specifically, when *Bsal *is lethal (*e.g.*, in *S. salamandra*), the staining intensity is highest, with relatively small variation (Fig. 4).

RCA 1 scores from the present study were also concordant with that from Wang et al.^33^, with the exception of the stratum corneum of *S. salamandra*, *P. waltl*, and *I. alpestris* (Supplementary Table 3; Supplementary Fig. 3). However, the relationship holds true for each different skin layer, including the stratum corneum (Supplementary Figure 4). Furthermore, none of the tested lectins showed a statistically significant correlation with the RCA 1 staining pattern (Supplementary Table 4).

#### Monoclonal antibodies

 In addition to the selected lectins, we further conducted a superficial exploration of the composition of potential *Bsal* receptors using antibodies targeting sulfated structures: (1) 6-sulfo LacNAc, (2) keratan sulfate, and (3) dermatan sulfate. Our observations revealed 6-sulfo LacNAc on the upper epidermal layers of only susceptible species and no reactivity in the resistant *L. helveticus* (Supplementary Fig. 1). Dermatan sulfate appeared absent in the skin of *S. salamandra*, while there was notable staining observed exclusively in dermal structures, excluding glandular contents, in *A. obstetricans *(Supplementary Figure 2A). Keratan sulfate was exclusively localised within the granular gland of *S. salamandra *while it was absent in *A. obstetricans *(Supplementary Fig. 2B).

### Correlation of RCA 1 with individual infection slopes

**Fig. 5:**
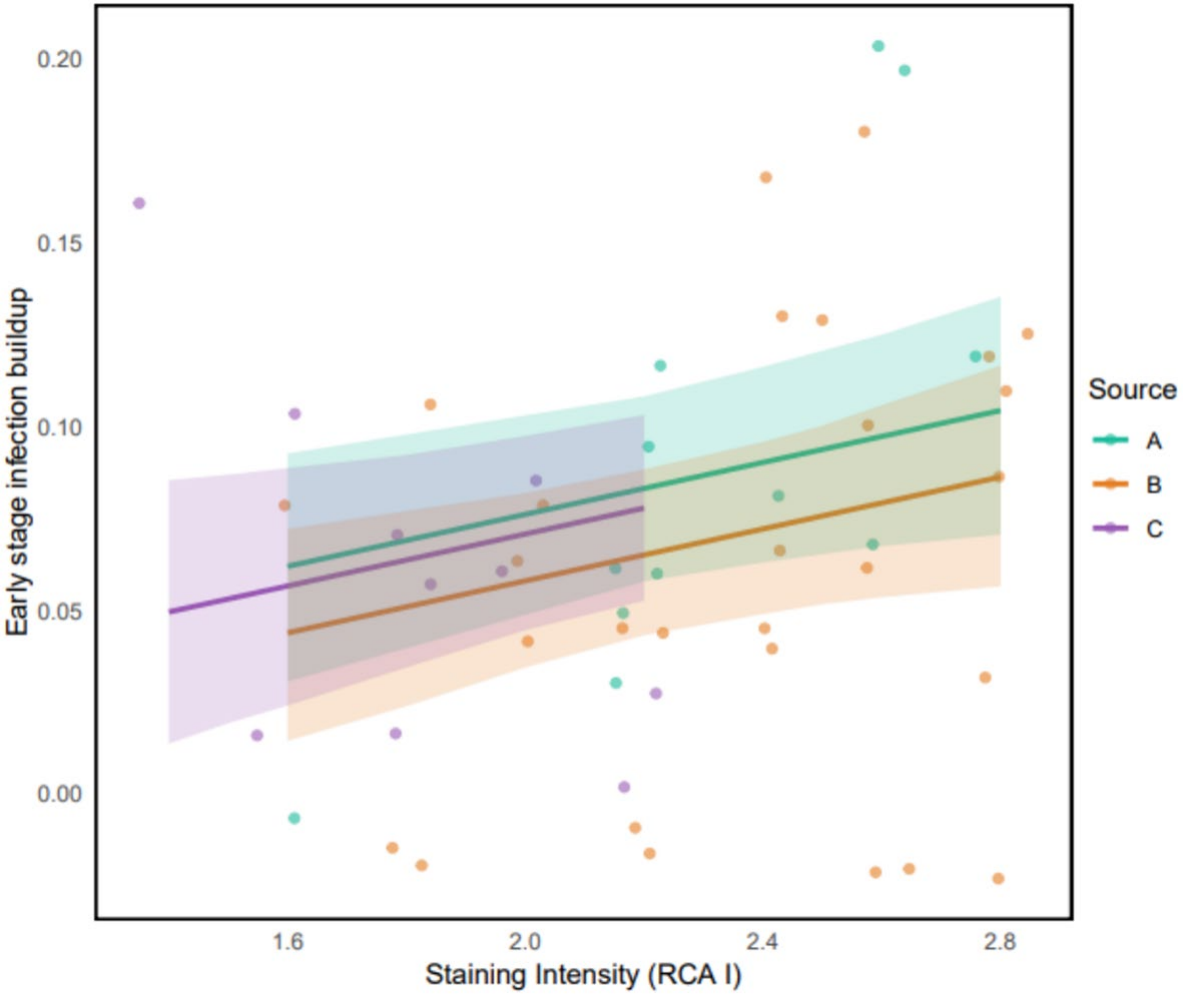
Correlation of RCA 1 staining intensity and early stage infection buildup. The regression lines (fitted using a linear mixed model) represent the trend between staining intensity and early stage infection buildup. Early stage infection buildup was measured as the log10-transformed peak pathogen load divided by the latency (time in weeks) to reach the peak within the first four weeks following *Bsal* exposure. Colours represent the different source populations. The shaded area represents the 95% confidence interval.

In the infection trial, *Bsal*-exposed *P. waltl* individuals began to test positive one week post-exposure while negative controls remained healthy with no detected pathogen load or clinical signs of chytridiomycosis throughout the experiment. Results showed early stage infection buildup was significantly influenced by epidermal glycosylation (F = 4.803, df = 38.161 *p*-value = 0.035) where newts with initial high RCA 1 scores experienced more intense *Bsal* infection after exposure (Fig. 5; Supplementary Table 5).

## Discussion

Our study investigated the binding patterns of various galactose-binding lectins and their potential association with species susceptibility to *Bsal*. By leveraging subtle differences in the lectins’ oligosaccharide specificity, a higher resolution of the components involved in *Bsal* adhesion may be achieved. We hypothesised that skin galactose plays a role primarily in the very early stages of infection. Thus selecting a narrow time frame of infection (i.e.*,* the rate to peak pathogen load within the first 4 weeks of infection) allows insights into the early dynamics of pathogenesis.

We demonstrated that only RCA 1 lectin histochemistry is a reliable marker of *Bsal* susceptibility trends across different species. Therefore, oligosaccharide structures recognised by PNA, ECL, and $\alpha$-galactose binding lectins may be ruled out as potential constituents of putative *Bsal* receptors. In particular, the results of ECL labelling were unexpected as the lectin was proposed as a replacement for RCA 1 due to their shared preference for type-II LacNAc^45^. However, this difference may be attributed to modifications of the terminal sugars. Investigations involving sulfated-galactan probes have shown that RCA 1 binding to Gal$\beta$1–4 is enhanced by 2-*O- *or 6-*O-*sulfation but abolished by 4-*O-*sulfation, contrasting with ECL, which cannot accommodate any sulfation on terminal $\beta$-gal^69^. Additionally, the inability of RPLs to replicate the patterns observed with their plant equivalents, despite specificity for the same monosaccharides, implies that specific oligosaccharide motifs are crucial for the correlation to susceptibility. We therefore conclude that the recombinant lectins do not function as ideal replacements for their plant ’equivalents’ in the context of this study.

Anti-6-sulfo LacNAc labelling showed that only susceptible species possess these structures on their skin, suggesting they may also serve as potential markers of susceptibility, independent of RCA 1-binding glycans. However, further investigation with resistant species is needed to confirm their functionality. Conversely, the complete absence of keratan and dermatan sulfate in the epidermis implies that other structures may be involved in the early dynamics of *Bsal* infection. It is important to note, that although RCA 1-binding glycans on the epidermis, likely involving LacNAc structures, may serve as target receptors for *Bsal*, glycomic analysis is needed to validate their exact composition and potential modifications.

The positive correlation of RCA 1 scores with early stage infection buildup suggests those with higher concentrations of epidermal galactose have weaker colonisation resistance in the early stages of infection due to the provision of more receptors for pathogen adhesion and proliferation. The epidermal galactose quantified by RCA 1 is thus a biomarker at the individual level that has important implications in marker-assisted selection—representing uses for phenotypic scoring of a quantitative trait (i.e., RCA 1-bound epidermal galactose) and ranking of individuals based on such merit. This approach offers a step toward an anti-adhesive strategy to enhance colonisation resistance in future generations of vulnerable populations, presenting an attractive conservation technique in reintroduction programs.

## Limitations and future directions

While our findings align with Wang et al.^33^, highlighting epidermal glycans as a critical early-stage infection factor, the process of pathogen attachment and invasion is likely more complex than simply a direct interaction between zoospores and epidermal galactose. In *B*d, mucins induce spore encystation^70^, but the role of specifically mucosal glycans in the context of *Bsal *infections remain unresolved^33^. Such an exploration would require a separate, more comprehensive study utilising ex vivo models and encystment assays which was beyond the scope of the experimental procedures employed in our current work. Particularly, paraffin clearing removes secreted mucins and lipids, precluding their quantification in our protocols^71^.

It remains an open question whether galactose expression is genetically controlled, environmentally influenced, or even diet-dependent. Investigating how metamorphic changes affect glycosylation may also be relevant, as glycan expression is species-, tissue-, and stage-specific^72^. Although, as previously mentioned, glycosylation patterns do not differ across body sites, receptor availability may vary with host age^30^.

## Conclusion

The results of our study demonstrate that only RCA 1-specific glycans may serve as a distinct biomarker for predicting *Bsal* susceptibility of amphibian species. We further conclude that RCA 1’s correlation with early stage infection buildup during the initial stages of infection illustrates the potential of exploiting the trait for successful intervention of pathogen binding. Anti-adhesive strategies through selective breeding or manipulation of the host glycome are critical in shielding the host from subsequent pathogen burden and deserve further investigation.

## Supplementary Information

Supplementary Information 1.

Supplementary Information 2.

Supplementary Information 3.

## Data Availability

All datasets generated or analysed during the current study are available upon request from the corresponding author.
