# Supplementary material for: RCA 1-binding glycans as a marker of Batrachochytrium salamandrivorans infection intensity at early stages of pathogenesis
Source: Sci Rep. 2025 Oct 28;15:37687. doi: 10.1038/s41598-025-21554-w (PMC12568929; doi:10.1038/s41598-025-21554-w)

**Supplementary Tables**

**Supplementary Table 1.**

| Skin layer | Species | RCA 1 (*SD*) | PNA (*SD*) | ECL (*SD*) | RPL-Gal1 (*SD*) | RPL-Gal4 (*SD*) |
| --- | --- | --- | --- | --- | --- | --- |
| Stratum corneum | *S. salamandra* | 2.31 (±0.38) | 0.71 (±1.19) | 0.08 (±0.20) | 0.02 (±0.05) | 1.60 (±0.62) |
|  | *P. waltl* | 0.58 (±0.52) | 0.00 (±0.00) | 0.00 (±0.00) | 0.56 (±0.39) | 1.48 (±0.41) |
|  | *I. alpestris* | 1.42 (±0.92) | 0.00 (±0.00) | 0.00 (±0.00) | 0.06 (±0.07) | 0.85 (±0.67) |
|  | *L. helveticus* | 0.46 (±0.37) | 0.13 (±0.21) | 0.08 (±0.20) | 0.10 (±0.20) | 0.56 (±0.30) |
| Stratum spinosum | *S. salamandra* | 2.56 (±0.55) | 0.25 (±0.61) | 0.13 (±0.31) | 0.89 (±0.27) | 0.92 (±0.52) |
|  | *P. waltl* | 1.21 (±1.02) | 0.08 (±0.13) | 0.33 (±0.49) | 1.44 (±0.53) | 0.92 (±0.47) |
|  | *I. alpestris* | 1.88 (±0.95) | 0.00 (±0.00) | 0.08 (±0.13) | 0.90 (±0.41) | 0.56 (±0.22) |
|  | *L. helveticus* | 0.54 (±0.46) | 0.00 (±0.00) | 0.38 (±0.44) | 0.85 (±0.26) | 0.58 (±0.40) |
| Stratum germinativum | *S. salamandra* | 2.75 (±0.29) | 0.38 (±0.92) | 0.46 (±0.46) | 0.91 (±0.30) | 1.15 (±0.45) |
|  | *P. waltl* | 1.13 (±0.82) | 0.08 (±0.13) | 0.33 (±0.44) | 1.63 (±0.50) | 1.06 (±0.49) |
|  | *I. alpestris* | 2.04 (±0.87) | 0.00 (±0.00) | 0.04 (±0.10) | 1.04 (±0.48) | 0.98 (±0.37) |
|  | *L. helveticus* | 0.54 (±0.46) | 0.00 (±0.00) | 0.08 (±0.13) | 0.92 (±0.26) | 0.63 (±0.37) |

**Supplementary Table 2.**

| Skin layer | Species | GSL 1 (*SD*) | RPL-Gal2 (*SD*) | RPL-Gal3 (*SD*) | RPL-αGal (*SD*) |
| --- | --- | --- | --- | --- | --- |
| Stratum corneum | *S. salamandra* | 1.85 (±0.46) | 0.54 (±0.78) | 0.84 (±0.48) | 0.08 (±0.13) |
|  | *P. waltl* | 2.03 (±0.57) | 0.54 (±0.33) | 1.54 (±0.81) | 0.25 (±0.35) |
|  | *I. alpestris* | 2.70 (±0.17) | 0.21 (±0.40) | 0.97 (±0.47) | 0.04 (±0.10) |
|  | *L. helveticus* | 1.38 (±0.40) | 0.29 (±0.37) | 0.63 (±0.23) | 0.25 (±0.61) |
| Stratum spinosum | *S. salamandra* | 1.90 (±0.46) | 1.04 (±0.66) | 2.23 (±0.41) | 0.75 (±0.42) |
|  | *P. waltl* | 2.01 (±0.34) | 1.08 (±0.68) | 1.85 (±0.44) | 0.80 (±0.69) |
|  | *I. alpestris* | 2.14 (±0.10) | 1.13 (±0.65) | 1.75 (±0.37) | 1.04 (±0.94) |
|  | *L. helveticus* | 2.44 (±0.17) | 1.29 (±0.75) | 1.43 (±0.41) | 1.13 (±0.49) |
| Stratum germinativum | *S. salamandra* | 2.37 (±0.13) | 1.21 (±0.68) | 2.00 (±0.43) | 0.75 (±0.42) |
|  | *P. waltl* | 2.64 (±0.40) | 1.33 (±0.89) | 2.23 (±0.49) | 0.80 (±0.69) |
|  | *I. alpestris* | 2.51 (±0.29) | 1.17 (±0.70) | 2.08 (±0.37) | 1.00 (±0.88) |
|  | *L. helveticus* | 2.72 (±0.18) | 1.33 (±0.80) | 1.81 (±0.46) | 1.33 (±0.70) |

**Supplementary Table 3.**

| Species | 2021 - 2024 Datasets | | |  | 2024 Dataset | | |
| --- | --- | --- | --- | --- | --- | --- | --- |
|  | Comparison | Z | *p*.adj |  | Comparison | Z | *p*.adj |
| *S. salamandra* |  |  |  |  | sg-ss | 0.440078 | 1 |
|  | 2021 - 2024sc | 3.500782 | 0.002319 |  | sg-mean | 0.831259 | 1 |
|  | 2021 - 2024ss | 2.068909 | 0.192773 |  | ss-mean | 0.391181 | 1 |
|  | 2021 - 2024sg | 1.542915 | 0.614256 |  | sg-sc | 1.638068 | 0.507037 |
|  | 2021 - 2024mean | 2.536459 | 0.05599 |  | ss-sc | 1.19799 | 1 |
|  |  |  |  |  | mean-sc | 0.80681 | 1 |
| *P. waltl* |  |  |  |  | sg-ss | -0.08466 | 1 |
|  | 2021 - 2024sc | 3.442457 | 0.002882 |  | sg-mean | 0.380987 | 1 |
|  | 2021 - 2024ss | 1.997763 | 0.228712 |  | ss-mean | 0.465651 | 1 |
|  | 2021 - 2024sg | 2.094076 | 0.181266 |  | sg-sc | 1.185293 | 1 |
|  | 2021 - 2024mean | 2.527484 | 0.057441 |  | ss-sc | 1.269957 | 1 |
|  |  |  |  |  | mean-sc | 0.804306 | 1 |
| *I. alpestris* |  |  |  |  | sg-ss | 0.427764 | 1 |
|  | 2021 - 2024sc | 3.162804 | 0.007813 |  | sg-mean | 0.745137 | 1 |
|  | 2021 - 2024ss | 2.063391 | 0.195378 |  | ss-mean | 0.317373 | 1 |
|  | 2021 - 2024sg | 1.569452 | 0.582714 |  | sg-sc | 1.379883 | 0.838113 |
|  | 2021 - 2024mean | 2.429862 | 0.075523 |  | ss-sc | 0.952119 | 1 |
|  |  |  |  |  | mean-sc | 0.634746 | 1 |
| *L. helveticus* |  |  |  |  | sg-ss | 0 | 1 |
|  | 2021 - 2024sc | 0.426169 | 1 |  | sg-mean | 0.161916 | 1 |
|  | 2021 - 2024ss | -0.20519 | 1 |  | ss-mean | 0.161916 | 1 |
|  | 2021 - 2024sg | -0.20519 | 1 |  | sg-sc | 0.53972 | 1 |
|  | 2021 - 2024mean | -0.01578 | 1 |  | ss-sc | 0.53972 | 1 |
|  |  |  |  |  | mean-sc | 0.377804 | 1 |

**Supplementary Table 4.**

|  | Estimate | 2.5% CI | 97.5% CI | t-value | df | Pr(\|t\|) |
| --- | --- | --- | --- | --- | --- | --- |
| (Intercept) | 2.059 | 0.616 | 3.508 | 2.585 | 23.151 | 0.017 |
| ECL | 0.706 | 0.12 | 1.288 | 2.199 | 49.178 | 0.033 |
| PNA | 0.117 | -0.946 | 1.187 | 0.199 | 49.136 | 0.843 |
| GSL 1 | -0.169 | -0.548 | 0.211 | -0.81 | 49.757 | 0.422 |
| RPL-Gal1 | -0.184 | -0.674 | 0.294 | -0.692 | 51.081 | 0.492 |
| RPL-Gal2 | 0.259 | 0.015 | 0.502 | 1.929 | 49.054 | 0.06 |
| RPL-Gal3 | -0.248 | -0.577 | 0.087 | -1.357 | 49.73 | 0.181 |
| RPL-Gal4 | 0.229 | -0.126 | 0.597 | 1.163 | 50.099 | 0.25 |
| RPL-αGal | 0.103 | -0.167 | 0.368 | 0.698 | 49.248 | 0.488 |
| Skin Layer [ss] | -0.148 | -0.528 | 0.235 | -0.707 | 49.132 | 0.483 |
| Skin Layer [sc] | -0.622 | -1.317 | 0.061 | -1.642 | 50.463 | 0.107 |

**Supplementary Table 5.**

|  | Estimate | 2.5% CI | 97.5% CI | t-value | df | Pr(\|t\|) |
| --- | --- | --- | --- | --- | --- | --- |
| (Intercept) | -0.035 | -0.554 | 0.483 | -0.134 | 33.323 | 0.894 |
| RCA 1 | 0.255 | 0.027 | 0.483 | 2.192 | 38.161 | 0.035* |

**Supplementary Figures**

**Supplementary Figure 1.**


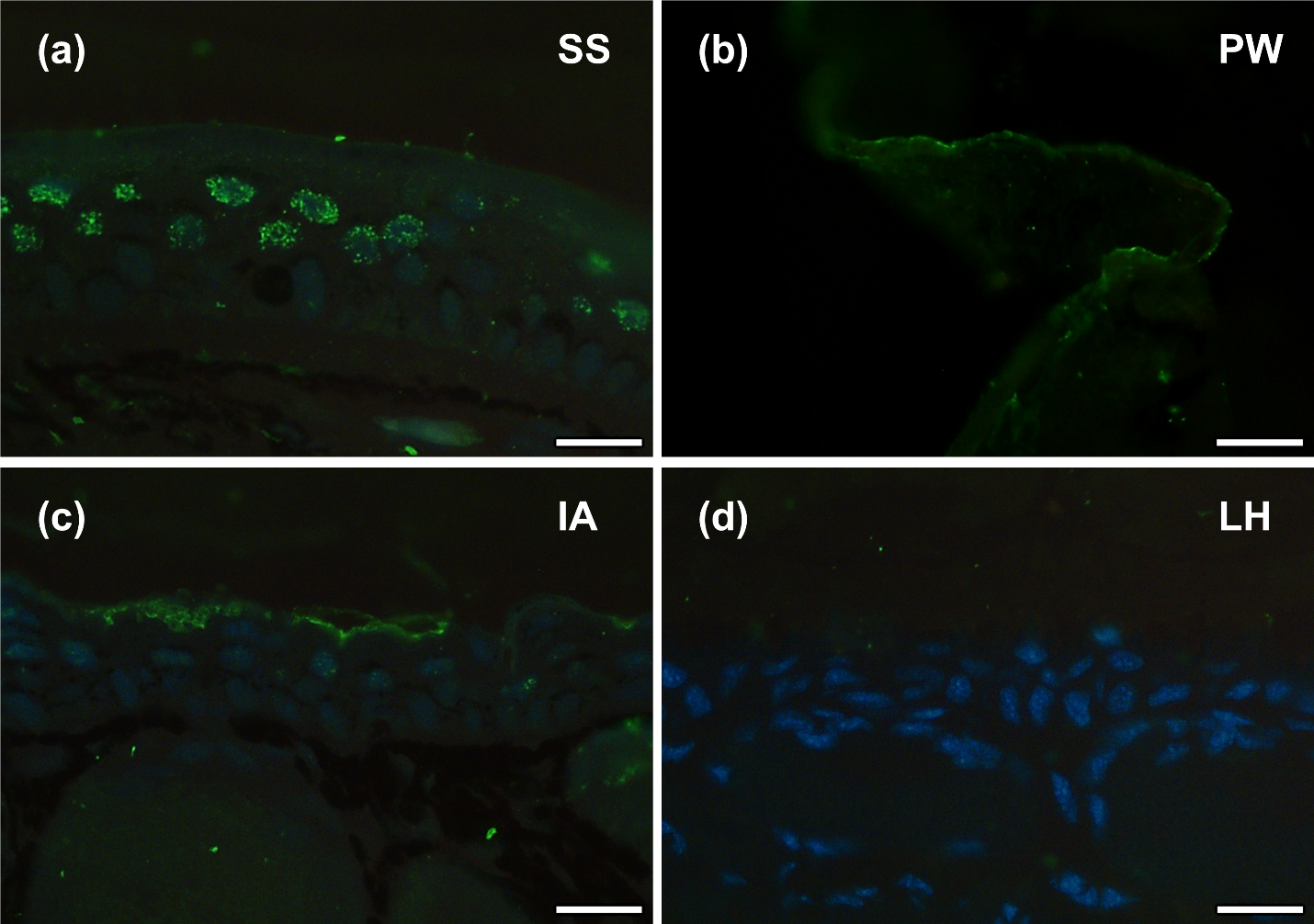


**Supplementary Figure 2.**


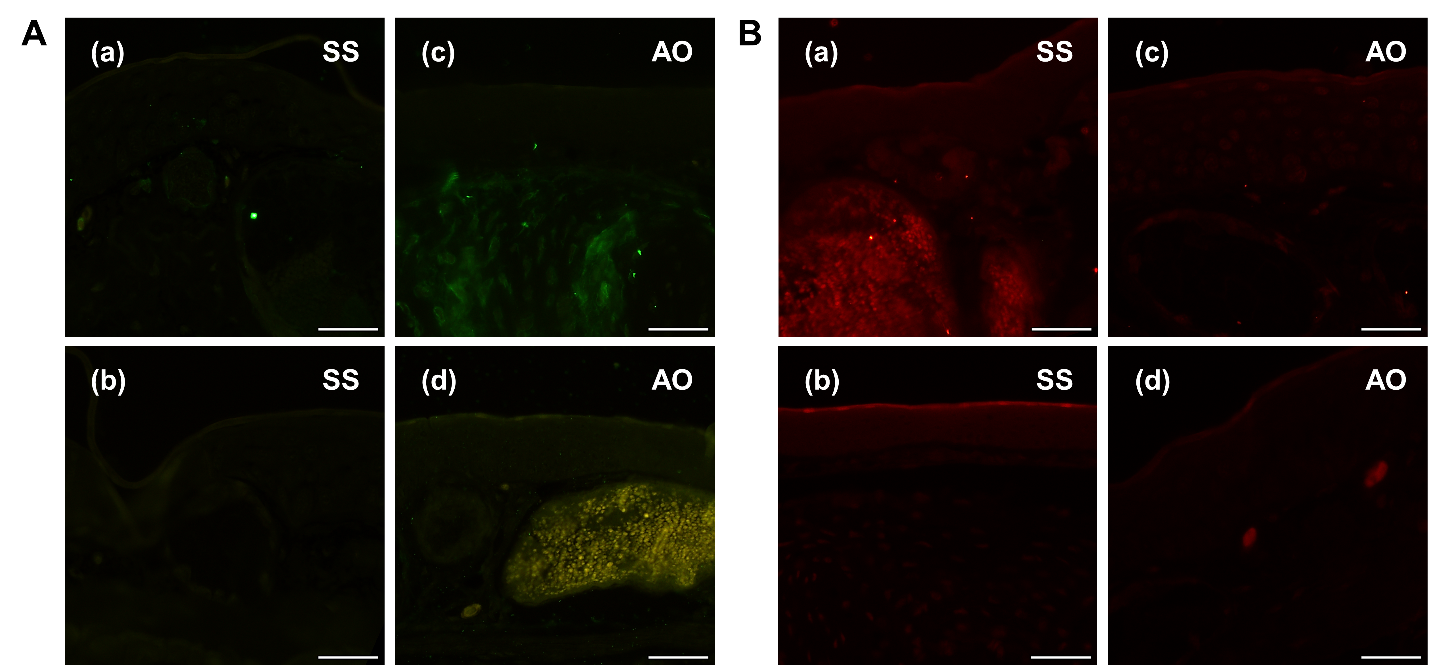


**Supplementary Figure 3.**


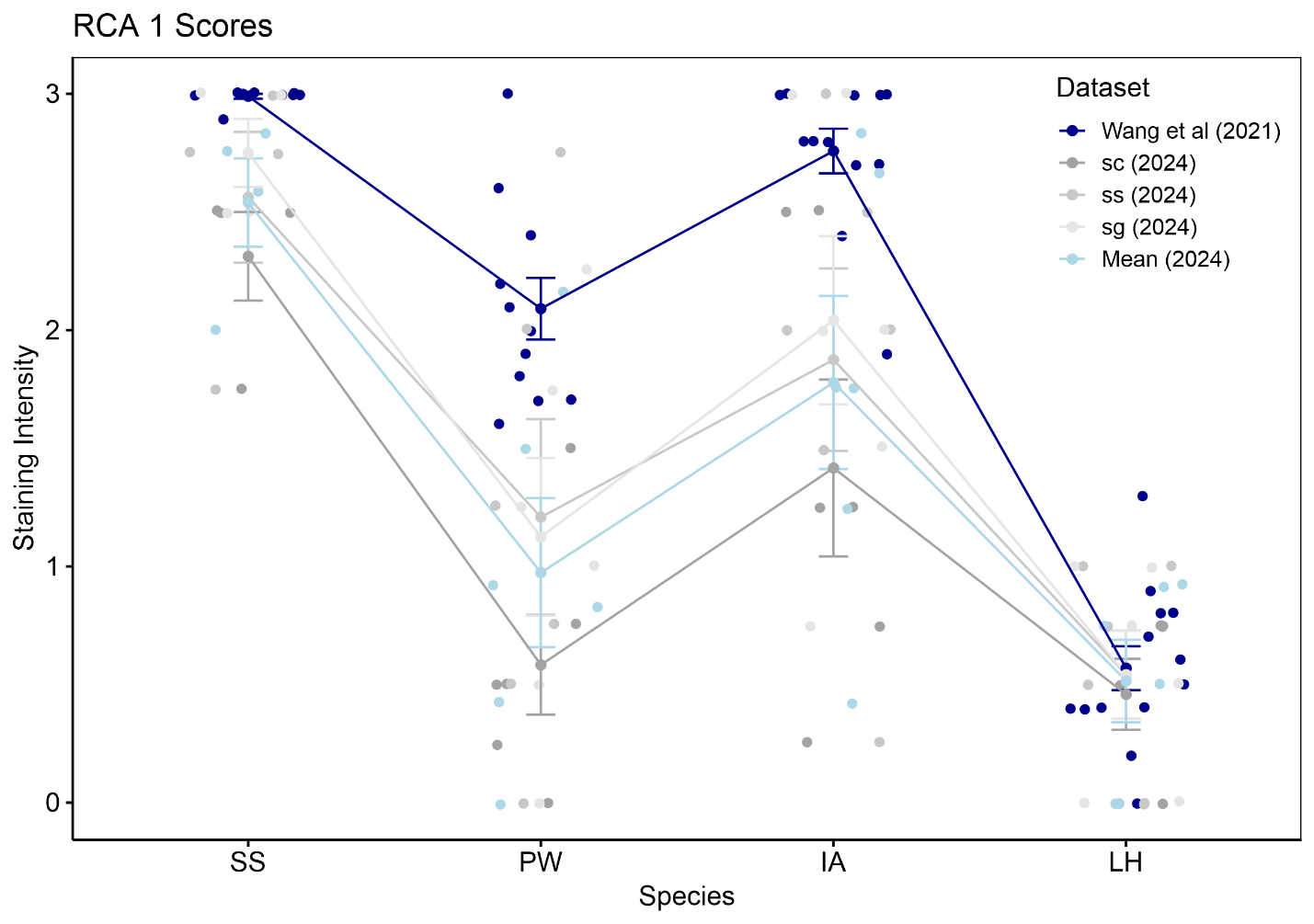


**Supplementary Figure 4.**


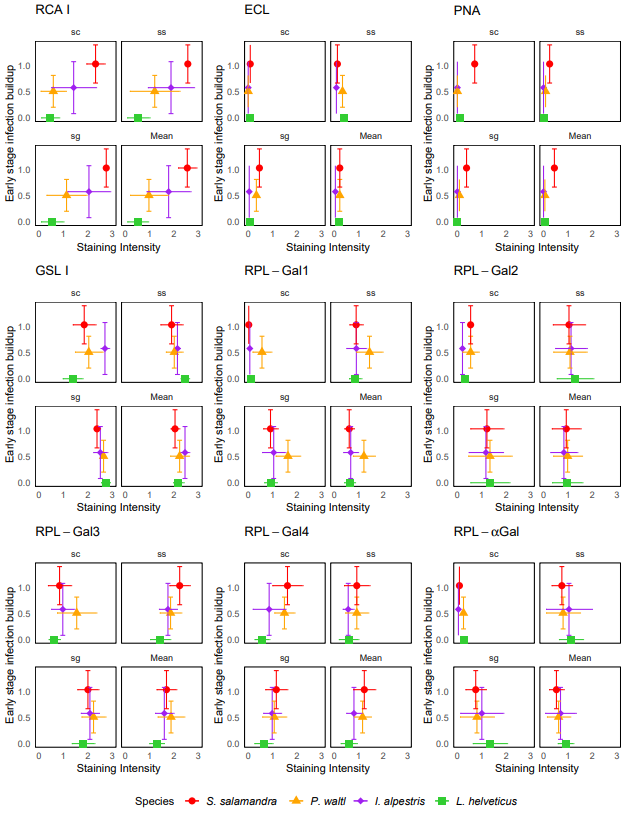

Supplement: Supplementary file 2 — Supplementary Information 2. [file 41598_2025_21554_MOESM2_ESM.docx]
