# Supplementary material for: RCA 1-binding glycans as a marker of Batrachochytrium salamandrivorans infection intensity at early stages of pathogenesis
Source: Sci Rep. 2025 Oct 28;15:37687. doi: 10.1038/s41598-025-21554-w (PMC12568929; doi:10.1038/s41598-025-21554-w)
Supplement: Supplementary file 3 — Supplementary Information 3. [file 41598_2025_21554_MOESM3_ESM.docx]

**Supplementary Tables**

**Supplementary Table 1. Mean scores of β-Gal binding lectins by skin layer and species**

Mean staining intensity scores (β -galactose-specific lectins) across skin layers by species. For each individual, staining intensity was calculated as the average of scores from four observers. Species-level means were then derived from these individual averages. Intraspecific variation is reported as standard deviation. Inter-observer variation is provided in the supplementary Excel file

**Supplementary Table 2. Mean scores of α-Gal binding lectins by skin layer and species**

Mean staining intensity scores (α-galactose-specific lectins) across skin layers by species. For each individual, staining intensity was calculated as the average of scores from four observers. Species-level means were then derived from these individual averages. Intraspecific variation is reported as standard deviation. Inter-observer variation is provided in the supplementary Excel file.

**Supplementary Table 3. Post-hoc Dunn’s test of RCA 1 scores of Wang et al. (2021) and the present study & RCA 1 scores of each skin layer**

Results of Dunn’s post-hoc test following a Kruskal–Wallis analysis of RCA 1 scores between Wang et al. (2021) and the present study. Abbreviations: sc = stratum corneum, ss = stratum spinosum, sg = stratum germinativum, mean = mean of the three epidermal strata, Z = Z values, *p*.adj = adjusted *p*-value.

**Supplementary Table 4. Results of linear mixed model analysis of RCA 1 with 8 lectins**

RCA 1 scores were regressed with the different lectin scores using a linear mixed model with the RCA 1 as the response variable, lectin scores and skin layer as the fixed effect, and species as the random effect. p-values were calculated using the Satterthwaite method. Note, after Bonferroni multiple correction (0.05/9 = 0.0055) there are no significant relationships. Abbreviations: sc = stratum corneum, ss = stratum spinosum

**Supplementary Table 5. Results of linear mixed model analysis of early stage infection buildup with RCA 1 staining intensity**

Individual RCA 1 scores from the infection trial were regressed with the early stage infection buildup index using a linear mixed model with the early stage infection buildup as the response variable, RCA 1 staining intensity scores as the fixed effect, and source population as the random effect. p-values were calculated using the Satterthwaite method.

**Supplementary Figures**

**Supplementary Figure 1. 6-sulfo LacNAc staining with DAPI overlay in blue indicating nuclei.** Note, the negative reaction with the epidermis in *L. helveticus.* Scale bar = 25 μm. Abbreviations: SS = *S. salamandra*, PW = *P. waltl*, LH = *L. helveticus*, IA = *I. alpestris*.

**Supplementary Figure 2. A. Dermatan sulfate labelling.** (a) Ventral skin of *S. salamandra* with (b) negative control ; (c) Toe clip of *A. obstetricans* with (d) negative control. Scale bar = 50 μm. **B. Keratan sulfate labelling.** (a) Ventral skin of *S. salamandra* with (b) negative control; (c) Toe clip of *A. obstetricans* with (d) negative control. Scale bar = 50 μm. Abbreviations: SS = *S. salamandra*, AO = *A. obstetricans*. Tissues stained only with the secondary antibody were used as the negative controls.

**Supplementary Figure 3. Comparison of RCA 1 scores of Wang et al. (2021) and the present study.** Abbreviations: sc = stratum corneum, ss = stratum spinosum, sg = stratum germinativum, Mean = mean of the three epidermal strata, SS = *S. salamandra*, PW = *P. waltl*, LH = *L. helveticus*, IA = *I. alpestris*.

**Supplementary Figure 4. Patterns of species mean lectin scores (individual skin layers) and early stage infection buildup.** Early stage infection buildup was measured as the log10-transformed peak pathogen load divided by the latency (time in weeks) to reach the peak within the first four weeks following *Bsal* exposure. Colours and symbols represent the different species. Abbreviations: sc = stratum corneum, ss = stratum spinosum, sg = stratum germinativum, Mean = mean of the three epidermal strata.
